# Essential points from Evidence-based Clinical Practice Guidelines for Chronic Kidney Disease 2018

**DOI:** 10.1007/s10157-018-1648-1

**Published:** 2018-12-01

**Authors:** Japanese Society of Nephrology, Hirokazu Okada, Yoshinari Yasuda, Naoki Kashihara, Koichi Asahi, Takafumi Ito, Shinya Kaname, Eiichiro Kanda, Yoshihiko Kanno, Kenichi Shikata, Yugo Shibagaki, Ken Tsuchiya, Kazuhiko Tsuruya, Daisuke Nagata, Ichiei Narita, Masaomi Nangaku, Motoshi Hattori, Takayuki Hamano, Shouichi Fujimoto, Toshiki Moriyama, Kunihiro Yamagata, Ryohei Yamamoto, Minako Wakasugi, Akira Ashida, Joichi Usui, Kazuko Kawamura, Kenichiro Kitamura, Tsuneo Konta, Yusuke Suzuki, Shuichi Tsuruoka, Saori Nishio, Takayuki Hamano, Naohiko Fujii, Hideki Fujii, Takehiko Wada, Hitoshi Yokoyama, Katsunori Aoki, Daiichiro Akiyama, Shin-ichi Araki, Hisatomi Arima, Eiji Ishikawa, Kenji Ishikura, Kiyonobu Ishizuka, Takuji Ishimoto, Yu Ishimoto, Kunitoshi Iseki, Mitsuyo Itabashi, Satoko Ichioka, Kazunobu Ichikawa, Daisuke Ichikawa, Shuji Inoue, Toshimi Imai, Hideaki Imamura, Yasunori Iwata, Yoshitaka Iwazu, Toshiaki Usui, Keiko Uchida, Masahiro Egawa, Shinichiro Ohara, Norio Omori, Rieko Okada, Yusuke Okuda, Takaya Ozeki, Yoko Obata, Hirayasu Kai, Noritoshi Kato, Keizo Kanasaki, Yoshikatsu Kaneko, Hideyuki Kabasawa, Takehiko Kawaguchi, Yukihiko Kawasaki, Keisuke Kawashima, Haruna Kawano, Kan Kikuchi, Masao Kihara, Yoshiki Kimura, Noriaki Kurita, Kentaro Koike, Masahiro Koizumi, Chiari Kojima, Shunsuke Goto, Takao Konomoto, Kentaro Kohagura, Hiroyuki Komatsu, Hirotaka Komaba, Chie Saito, Yukinao Sakai, Yusuke Sakaguchi, Hiroshi Satonaka, Kanako Jimi, Akihiro Shimizu, Sayaka Shimizu, Sayuri Shirai, Maki Shinzawa, Kazuhiro Sugiyama, Tomo Suzuki, Hitoshi Suzuki, Kazuhide Suyama, Hiroyoshi Segawa, Kazuya Takahashi, Kenichi Tanaka, Tetsuhiro Tanaka, Ryoya Tsunoda, Yuki Tsuruta, Hyogo Nakakura, Yasuyuki Nagasawa, Koichi Nakanishi, Masahiko Nagahama, Izaya Nakaya, Masayoshi Nanami, Kakuya Niihata, Shinichi Nishi, Hiroki Nishiwaki, Shoko Hasegawa, Midori Hasegawa, Ken Hanada, Hiroki Hayashi, Ryoko Harada, Manabu Hishida, Daishi Hirano, Junichi Hirahashi, Akio Hirama, Kouichi Hirayama, Masafumi Fukagawa, Akihiro Fukuda, Yoshiyuki Fujii, Kiichiro Fujisaki, Fumihiko Furuya, Junichi Hoshino, Michihiro Hosojima, Kenjiro Honda, Takahiro Masuda, Kosuke Matsui, Yuta Matsukuma, Hideki Matsumura, Akiko Mii, Kenichiro Miura, Michihiro Mitobe, Yoshikazu Miyasato, Satoshi Miyamoto, Saori Miwa, Masahiko Yazawa, Yusuke Yata, Yoshihiro Yamamoto, Kimio Watanabe

**Affiliations:** Nichinai-Kaikan 6F, 3-28-8 Hongo, Bunkyo-ku, Tokyo, 113-0033 Japan

## Levels of evidence

A High: We are confident that the true effect lies close to that of the estimate of the effect.

B Moderate: The true effect is likely to be close to the estimate of the effect, but there is a possibility that it is substantially different.

C Low: The true effect may be substantially different from the estimate of the effect.

D Very low: The estimate of the effect is very uncertain and might often be far from the true effect.

None

## Grade of recommendation

1. “We recommend”
2. “We suggest”

None

## Chapter 1. Diagnosis and definition of chronic kidney disease

CQ1-1: How can we diagnose CKD?

Statement: CKD is defined as the presence of either of the conditions listed below lasting for more than 3 months. (Level: None, Grade: 1)

1. Findings suggesting kidney damage, i.e., abnormal findings in blood or urinary tests, imaging studies or pathological evaluations. In particular, evidence of proteinuria ≥ 0.15 g/gCr (albuminuria ≥ 30 mg/gCr) is important.
2. GFR < 60 mL/min/1.73 m^2^

In clinical practice, eGFR is calculated by the following GFR equation adjusted for the Japanese:

eGFR (mL/min/1.73 m^2^) = 194 x Cr^-1.094^ x Age^-0.287^ (x 0.739 if female)

Note: We recommend that serum creatinine (Cr) value (mg/dL) should be evaluated by the enzymatic assay method and rounded off to 2 decimal places. The Japanese GFR equation is applicable to adults aged 18 years or older.

CQ 1–2: How can we evaluate the severity of CKD?

Statement: We recommend that CKD severity should be evaluated by cause, GFR category, and degree of proteinuria/albuminuria based on the CGA classification. (Level: A, Grade: 1)

CQ 2: Should routine health check-up patients with dipstick proteinuria (1+ or greater) be referred for clinic consultation?

Statement: In health check-up subjects, dipstick proteinuria (1 + or greater) is a significant risk factor not only for ESKD but also for CVD and all-cause mortality. We recommend that health check-up subjects with dipstick proteinuria (1+ or greater) consult a specialized clinic, because these risks could be decreased by adequate treatment. (Level: C, Grade: 1)

CQ 3: Should the elderly (aged ≥ 65 years) routine health check-up subjects with an eGFR < 45, be recommended to visit the clinic?

Should routine health check-up patients with dipstick proteinuria (1+ or greater) be referred for clinic consultation?

Statement: Even among the elderly, aged ≥ 65 years, the risks of all-cause mortality and ESKD risks increased in those with an eGFR level < 45. Thus, we recommend that elderly CKD patients with an eGFR level of less than 45 should visit the clinic. (Level: B, Grade: 1)

CQ 4: Is examination of proteinuria/albuminuria recommended in the Specific Health Check-ups and Guidance Programs in Japan?

Statement: Examination of proteinuria/albuminuria is recommended in the Specific Health Check-ups and Guidance Program in Japan, because proteinuria/albuminuria is associated with increased risks of all-cause mortality, CVD onset, and renal impairment. (Level: C, Grade: 1)

## Chapter 2. Modification of life-style

Q1: Is smoking cessation recommended for people with CKD?

Statement: We recommend that individuals with CKD stop smoking to reduce progression of CKD and risk of all-cause mortality. (Level: B, Grade: 1)

CQ2: How much alcohol is safe for patients with CKD?

Statement: There is insufficient evidence to recommend an adequate amount of alcohol to adults with CKD. (Level: D, Grade: None)

CQ3: Are treatments for sleep apnea syndrome (SAS) recommended for patients with CKD?

Statement: We suggest that CKD patients with SAS undergo medical treatment. (Level: D, Grade: 2)

CQ4: Are pneumococcal and influenza vaccination recommended for patients with CKD?

Statement: We suggest that patients with CKD should be vaccinated because both vaccines induce higher titers of antibodies. Clinical benefits of these vaccines in preventing infections remains to be elucidated. (Level: C, Grade: 2)

## Chapter 3. Management of diet

CQ1: Is the intervention of a registered dietitian recommended for CKD patient treatment?

Statement: We recommend intervention of registered dietitian with specific training on medical guidance of CKD patients to prevent the progression of CKD stages. (Level: C, Grade: 1)

CQ2: Is dietary protein restriction recommended to prevent the progression of CKD?

Statement: We recommend individualized protein restriction for patients with CKD in accordance with their specific clinical condition, in addition to nutrition guidance consisting of a low protein diet under the management of the medical team with nephrologists and registered dietitians. (Level: B, Grade: 1)

CQ3: Should serum potassium of patients with CKD be corrected for the reduction of mortality and CVD?

Statement: We suggest that serum potassium levels should be maintained between 4.0 and 5.4 mEq/L for the reduction of mortality and CVD in patients with CKD. (Level: C, Grade: 2)

CQ4: Is it recommended to restrict salt intake between 3 and 6 g/day for better prognosis of CKD patients?

Statement: We recommend restricting salt intake to below 6 g/day to prevent hypertension, proteinuria, and CVD. It is recommended to set a lower limit for each patient with 3 g/day as a guide because extreme salt restriction could be harmful. (Level: C, Grade: 1)

CQ5: Is it recommended to treat metabolic acidosis of CKD patients to prevent deterioration of renal function?

Statement: We recommend treating metabolic acidosis with sodium bicarbonate and other such bases to prevent hypertension, proteinuria, and CVD. It is recommended to consider treatment when a patient’s HCO_3_^-^ is below 21 mmol/L. (Level: B, Grade: 1)

## Chapter 4. Management of hypertension and cardiovascular disease

CQ1: Could appropriate BP control prevent the incidence of CKD in hypertensive patients?

Statement: There is substantial supportive evidence indicating a positive association between high BP and development of incident CKD. Management of hypertension is strongly recommended for the prevention of CKD. Treatment to control excessive BP variations, such as nocturnal hypertension or morning surge, might be more beneficial. (Level: C, Grade: 1)

CQ2: Is a target BP < 130/80 mmHg recommended for CKD patients (< 75 years of age) associated with hypertension?

Statement:

<CKD stage G1, 2>

A target BP < 130/80 mmHg is recommended for adults with hypertension and CKD associated with diabetes. (Level: B, Grade: 1)

A target BP < 140/90 mmHg is recommended for adults with hypertension and CKD with A1 level proteinuria not associated with diabetes. (Level: A, Grade: 1)

A target BP < 130/80 mmHg is recommended for adults with hypertension and CKD with A2 or A3 level proteinuria not associated with diabetes. (Level: C, Grade: 1)

<CKD stage G3–5>

A target BP < 130/80 mmHg is recommended for adults with hypertension and CKD associated with diabetes. (Level: C, Grade: 2)

A target BP < 140/90 mmHg is recommended for adults with hypertension and CKD with A1 level proteinuria not associated with diabetes. (Level: C, Grade: 2)

A target BP < 130/80 mmHg is recommended for adults with hypertension and CKD with A2 or A3 level proteinuria not associated with diabetes. (Level: C, Grade: 2)

A systolic BP suppression below 110 mmHg is not recommended for adults with hypertension and CKD, regardless of CKD stage. (Level: C, Grade: 2)

CQ3: Is a target BP < 150/90 mmHg recommended for elderly CKD patients (aged ≥ 75 years old) associated with hypertension?

Statement:

<CKD stage G1, 2>

A target BP < 150/90 mmHg is recommended for elderly patients with hypertension and CKD. (Level: C, Grade: 1)

A target BP < 140/90 mmHg is recommended for elderly patients with hypertension and CKD who may tolerate treatment with antihypertensive agents without any adverse events. (Level: C, Grade: 1)

<CKD stage 3–5>

Recommendations are the same as those for CKD stage G1, 2. (Level: C, Grade: 2)

Systolic BP suppression below 110 mmHg is not recommended for elderly patients with hypertension and CKD, regardless of the CKD stage. (Level: C, Grade: 2)

CQ4: What is the appropriate antihypertensive agent for CKD patients associated with hypertension?

Statement:

In CKD patients with diabetes mellitus (A1–A3 category) and without diabetes mellitus (A2, 3 category), ACEIs or ARBs are recommended. (Level: B, Grade: 1)

In CKD patients without diabetes (A1 category), ACEIs, ARBs, CCBs or thiazide diuretics are recommended. (Level: B, Grade: 1)

In CKD stages G4, 5, CCBs are recommended if ACEIs or ARBs may not be tolerated, despite dose-reduction, due to worsening of renal function or hyperkalemia. (Level: C, Grade: 1)

In CKD stages G4, 5 in elderly patients aged more than 75 years, initial therapy should be CCBs because RAS inhibitors could deteriorate renal function in elderly patients due to dehydration or ischemia. (Level: C, Grade: 1)

CQ5: What are the appropriate antihypertensive agents for adults with CKD and CVD?

Statement: Recommended antihypertensive agents and the evidence levels of their prescriptions depend on the categories of CVD and the stage of CKD without stage 5D and transplantation.

<CKD stage G1-3a>

[Coronary artery disease (CAD)]

Initial antihypertensive therapy should be ACEIs, beta-blockers, or ARBs. (Level: A, Grade: 1)

[Heart failure with reduced ejection fraction (HFrEF)]

ACEIs, beta-blockers, MRBs, or ARBs are recommended as the preferred antihypertensive agents in patients with CKD and HFrEF. (Level: A, Grade: 1)

[Heart failure with preserved ejection fraction (HFpEF)]

Initial antihypertensive therapy might consist of beta-blockers, ACEIs, ARBs, or MRBs. (Level: C, Grade: 2)

[Volume overload]

Initial antihypertensive therapy might consist of diuretics. (Level: D, Grade: None)

[Stroke (chronic phase), Peripheral artery diseases]

Appropriate initial antihypertensive agents could not be recommended. (Level: D, Grade: None)

<CKD Stage G3b-5 without G5D and transplantation>

[Coronary artery disease (CAD)]

Initial antihypertensive therapy should be ACEIs, beta-blockers, or ARBs. (Level: C, Grade: 2) (Table 1)

**Table 1:**
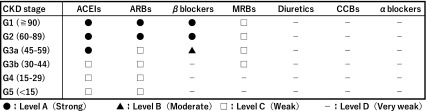
In case of CKD with coronary artery disease

[Heart failure (HFrEF)]

ACEIs or ARBs are recommended as the preferred antihypertensive agents in patients with CKD and HFrEF. (Level: D, Grade: 2) (Table 2)

**Table 2:**
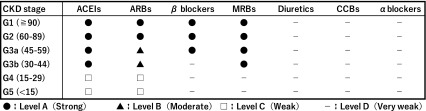
In case of CKD with heart failure (HFrEF)

[Heart failure (HFpEF)]

Initial antihypertensive therapy may consist of ACEIs or ARBs. (Level: D, Grade: 2)

[Volume overload]

Initial antihypertensive therapy may consist of diuretics. (Level: D, Grade: None)

[Stroke (chronic phase), Peripheral artery diseases]

Appropriate initial antihypertensive reagents could not be recommended. (Level: D, Grade: None)

In CKD patients stage G3b-5 (without 5D and transplant), antihypertensive therapy with ACEIs or ARBs might be intolerable due to hyperkalemia or renal impairment. The initial dose of ACEIs or ARBs should be low and gradually titrated upward as needed. (Level: D, Grade: None)

## Chapter 5. Management of nephrosclerosis and renal artery stenosis

CQ1: Is strict reduction of BP recommended for CKD caused by nephrosclerosis with hypertension?

Statement: We do not suggest the strict reduction of BP to a systolic pressure of < 120 mmHg in CKD caused by nephrosclerosis with hypertension, especially in A1 category proteinuria due to the risk of AKI. We suggest a target level of < 140/90 mmHg. (Level: C, Grade: 2)

CQ2: Which antihypertensive drugs are recommended for CKD with renal artery stenosis?

Statement: We suggest the use of RAS inhibitor for CKD with unilateral renal artery stenosis because it is superior in reducing BP in such patients compared to other antihypertensive drugs and may suppress progression to mortality, onset of CVD and progression of renal dysfunction. However, to avoid the risk of AKI, it is necessary to adjust the dose carefully starting with a low dose and checking the serum Cr and K levels approximately 2 weeks after start. A RAS inhibitor is not used in principle when bilateral renal artery stenosis is suspected. (Level: C, Grade: 2)

CQ3: Which imaging studies are recommended for CKD (Stage G1-3) with suspected renal artery stenosis?

Statement: We suggest performing renal artery ultrasound as a screening test first, followed by plain MR angiography. When CT angiography and gadolinium-enhanced MR angiography are performed, the risk of contrast-induced nephropathy and nephrogenic systemic fibrosis must be considered carefully. When these tests do not lead to a diagnosis or when the indication of angioplasty is considered, we suggest performing renal artery angiography. (Level: C, Grade: 2)

CQ4: Is revascularization recommended for CKD with arteriosclerotic renal artery stenosis?

Statement: We do not suggest performing revascularization for CKD with arteriosclerotic renal artery stenosis in principle due to the risk of complications, because it does not suppress the progression of renal dysfunction or decrease the risk of developing CVD. (Level: B, Grade: 2)

## Chapter 6. Renal anemia

CQ1: What is the target Hb range in prescribing ESA for renal anemia in CKD patients?

Statement: We propose that the target Hb range for prescribing ESA to non-dialysis CKD patients is 11 g/dL or more and below 13 g/dL. In case of ESA hypo-responsiveness, determining the cause and correcting it are mandatory. Attention should be paid to avoiding over-dosage of ESA. For patients with a past history or complication of severe CVD or any other medical indications, one should consider reducing or terminating ESA use if Hb level exceeds 12 g/dL (Level: B, Grade: 2).

CQ2: Is iron treatment recommended in anemic CKD patients with iron deficiency?

Statement: We propose iron treatment for anemic CKD patients with iron deficiency. (Level: B, Grade: 2)

## Chapter 7. Management of chronic kidney disease-mineral bone disease

CQ1: Is administration of phosphate binders recommended in non-dialysis CKD patients with hyperphosphatemia?

Statement: In non-dialysis CKD patients with hyperphosphatemia, the use of phosphate binders may be considered because phosphate binders have the possibility of reducing progression to mortality. (Level: C, Grade: 2)

CQ2: Is the administration of non-calcium-containing phosphate binders recommended for the treatment of hyperphosphatemia in non-dialysis CKD patients?

Statement: In non-dialysis CKD patients with hyperphosphatemia, the administration of non-calcium-containing phosphate binders may be considered because they are potentially more effective in preventing progression to mortality and vascular calcification progression compared to calcium-containing phosphate binders (Level: C, Grade: 2).

CQ3: Is administration of active vitamin D recommended in non-dialysis CKD patients?

Statement: In non-dialysis CKD patients, administration of active vitamin D may be considered because it lowers PTH levels and may reduce proteinuria. However, active vitamin D can cause hypercalcemia and it is unknown whether this therapy decreases the risk of CKD progression, fracture, cardiovascular events, or mortality. Thus, we suggest therapeutic decisions be made on a case-by-case basis and active vitamin D be started with small doses. We also suggest that, in the case of hypercalcemia or worsening renal function, active vitamin D should be reduced or stopped promptly. (Level: C, Grade: 2)

CQ4: Is administration of anti-osteoporotic agents recommended in non-dialysis CKD patients?

Statement: In non-dialysis CKD patients, administration of anti-osteoporotic agents, such as active vitamin D, bisphosphonates, selective estrogen receptor modulators (SERMs), teriparatide, and denosumab may be considered because these may reduce fracture risk. Special attention, however, needs to be paid during treatment in CKD patients, because, depending on the agent, the expected beneficial and adverse effects may differ from those observed in the general population. (Level: D, Grade: 2)

## Chapter 8. Management of hyperuricemia and dyslipidemia

CQ1: Is uric acid-lowering therapy recommended in patients with CKD?

Statement: Uric acid-lowering therapy is suggested for patients with CKD and hyperuricemia, because it may potentially slow the progression of renal dysfunction and reduce urinary protein excretion. (Level: C, Grade: 2)

CQ2: Is lipid-lowering therapy recommended in patients with CKD?

Statement: Lipid-lowering therapy with statins or statins plus ezetimibe is recommended for patients with CKD and dyslipidemia, because it may potentially inhibit the development and recurrence of CVD events, reduce urinary protein excretion, and slow progression of renal dysfunction (Level: B Grade: 2). Meanwhile, lipid-lowering therapy with fibrates also could be useful for preventing development and recurrence of CVD events in those patients; however, we should use them carefully because fibrates are contraindicated or necessitate to be administered carefully in patients with moderate to severe renal dysfunction (Level: D, Grade: None).

## Chapter 9. Management of metabolic syndrome

CQ1: Are obesity and metabolic syndrome risk factors of mortality, CVD, ESKD, and progression of CKD in patients with CKD?

Statement: Metabolic syndrome is potentially associated with mortality, CVD, ESKD, and progression of CKD in patients with CKD, although obesity is not obviously associated with these outcomes. (Level: C, Grade: None)

CQ2: Is exercise training recommended for CKD patients with obesity and/or metabolic syndrome?

Statement: We suggest that CKD patients with obesity and/or metabolic syndrome follow an aerobic exercise training program, which is effective for weight loss and improvement of peak oxygen consumption (VO_2_ peak). Considering the clinical characteristics of each patient, the adequate load of aerobic exercise training should be estimated carefully. (Level: C, Grade: 2)

## Chapter 10. Management of pregnancy

CQ1: Do CKD patients have higher risks of pregnancy complications (i.e., for preeclampsia, premature birth, or fetal loss?

Statement: CKD patients have higher risks of pregnancy complications, such as preeclampsia, premature birth, or fetal loss, even in the CKD stage of G1. The risk is higher in patients with a higher CKD stage. (Level: C, Grade: 1)

CQ2: What antihypertensive agents are recommended for pregnant hypertensive CKD patients?

Statement: When antihypertensive medications are necessary, not only in CKD patients, we can use methyldopa, labetalol, hydralazine, and slow-release nifedipine after the 20th week of pregnancy. ACEIs and ARBs are contraindicated in pregnant women. (Level: D, Grade: 2)

## Chapter 11. Management of pediatric CKD

CQ1: Is urinary screening recommended for children at 3 years of age and throughout the school age?

Statement: In pediatric populations, urinary screening at 3 years of age and throughout school age is considered to contribute to the discovery of CKD at an early stage, thus, facilitating early management and improving the renal prognosis. (Level: D, Grade: 2)

CQ2: Is CKD in pediatric patients a risk factor for CVD?

Statement: CKD in pediatric patients is a risk factor for CVD. (Level: C, Grade: None)

CQ3: Should low birth weight, preterm birth, and fetal growth retardation be treated as risk factors for CKD?

Statement: Since low birth weight, preterm birth, and fetal growth retardation are associated with future onset of CKD, it is recommended that they should be treated as risk factors. (Level: B, Grade: 1)

CQ4: Is exercise recommended for pediatric patients with CKD?

Statement: We suggest that pediatric patients with CKD perform mild to moderate exercise to improve QOL, motor function, and respiratory function. (Level: C, Grade: 2)

CQ5: Is protein intake restriction recommended for pediatric patients with CKD?

Statement: As any preventive effects due to protein intake restriction against deterioration of renal function are still unclear, we suggest that this should not be implemented. (Level: B, Grade: 2)

CQ6: Is vaccination recommended for pediatric patients with CKD?

Statement: As pediatric patients with CKD are susceptible to infections that are likely to become severe, vaccination is recommended. (Level: C, Grade: 2)

CQ7: Is antihypertensive therapy recommended for pediatric CKD patients with hypertension?

Statement: For pediatric CKD patients with hypertension we suggest that antihypertensive therapy should be implemented along with lifestyle instruction and medication including RA inhibitors and calcium antagonists, on an individual basis and according to age. (Level: C, Grade: 2)

CQ8: Is management and therapy for CKD-MBD recommended for pediatric patients with CKD?

Statement: As management of CKD-MBD for pediatric patients with CKD may improve bone lesions and cardiovascular function, we recommend that it should be implemented. We suggest that the corrected serum levels of calcium and phosphorus should be maintained within normal ranges in accordance with patient age. (Level: C, Grade: 2)

CQ9: Is administration of recombinant human growth hormone (rhGH) recommended for pediatric patients with CKD showing growth stunting?

Statement: We suggest that rhGH administration should be considered for pediatric CKD patients with growth stunting as it can significantly increase the height of patients. (Level: A, Grade: 2)

CQ10: Is preemptive kidney transplantation (PEKT) recommended for pediatric patients with CKD?

Statement: We suggested that preemptive kidney transplantation should be considered for pediatric patients with CKD, as the likelihood of graft survival is higher than that after dialysis has been implemented. (Level: C, Grade: 2)

## Chapter 12. Management of elderly CKD

Preamble: Renal replacement therapy (RRT) for elderly CKD patients aged more than 75 years

In elderly CKD patients aged more than 75 years, an improvement in life prognosis by RRT can be expected. It is important to consult with nephrologists/special medical institutions when choosing RRT. The selection should be based on appropriate decision-making through discussion with the patients and their families by comprehensive consideration through the evaluation of renal function, medical history, multiple complications (CVD, malignant neoplasms, advanced dementia, severe frailty, and other), the patient’s social situation, and anticipated life expectancy. In addition, understanding and practice of an appropriate preservation therapy and palliative care are required when the patients decide to forgo hemodialysis.

CQ1: Is treatment for CKD-MBD recommended for the elderly (≥ 75 years) CKD patients?

Statement: In elderly patients (aged 75 years or more) with non-dialysis CKD, we suggest limiting dietary phosphate intake and/or using phosphate binders to maintain serum phosphate levels within the normal range. In this population, close monitoring for adverse effects is required to prevent malnutrition since the phosphate-lowering therapies may cause loss of appetite. (Level: C, Grade: 2)

In elderly non-dialysis CKD patients with secondary hyperparathyroidism, we suggest lowering parathyroid hormone levels within the normal range by treating abnormalities in serum phosphate and calcium levels before administering active vitamin D agents. Hypercalcemia must be avoided when using active vitamin D agents. (Level: C, Grade: 2)

CQ2: Can intervention in elderly CKD patients aged more than 75 years, prevent or control the progression of frailty, improve life prognosis, prolong induction of renal replacement therapy?

Statement: In elderly CKD patients aged more than 75 years, frailty is one of the indicators of renal and life prognoses and incidence of renal replacement therapy. The effectiveness of intervention, nutrition therapy and exercise, in frail individuals is still not known. (Level: D, Grade: None)

CQ3: Is lipid lowering therapy recommended for elderly CKD patients aged more than 75 years?

Statement: For elderly CKD patients aged 65–71 years, we propose that lipid lowering therapy (statin alone or a statin and ezetimibe combination) should be performed in elderly CKD patients aged 75 years or more since statins are effective for decreasing all -cause mortality and for primary or secondary prevention of CVD. (Level: C, Grade: 2)

CQ4: Is renal biopsy recommended for elderly people aged more than 75 years?

Statement: There is no basis for contraindications of renal biopsy in elderly individuals aged > 75 years. There is also the possibility that the appropriate treatment can be determined based on renal biopsy. With respect to implementation, renal biopsy on elderly people should be introduced to experienced nephrologists and specialized medical institutions and their recommendations should be judged in consideration of renal function prognosis/life prognosis. (Level: D, Grade: None)

CQ5: Could strict glycemic control (HbA1c < 7.0%) be recommended for CKD patients with DM aged more than 75 years?

Statement: CKD patients with DM aged ≥ 75 years should be recognized as being at risk of severe hypoglycemia and falls with strict glycemic therapy. We suggest that the target for glycemic control for elderly CKD patients should be decided individually depending on the health condition (age, cognitive function, physical function, comorbidity, risk of hypoglycemia, and time to live). (Level: D, Grade: None)

CQ6: What is the optimal hemoglobin target range for anemic elderly patients (≥75 years) CKD patients?

Statement: In anemic elderly patients (aged ≥ 75 years) with non-dialysis CKD, we suggest maintaining hemoglobin levels within the target range of 11–13 g/dL using erythropoietin-stimulating agents (ESAs) and/or iron supplementation. With respect to the mortality risk, the lower limit of the target range could be lowered to 9 g/dL. During the ESA therapy, clinical factors associated with resistance to ESAs should be removed to avoid use of high-dose ESAs. (Level: C, Grade: 2)

CQ7: Are there any drugs to be especially attentive on drug usage for elderly CKD patients aged more than 75 years in daily clinical practice?

Statement: Metabolic and excretion rates decline in elderly CKD patients aged more than 75 years, and attention must be paid to the control of the doses of RAS inhibitors, diuretics, vitamin D, and other drugs, which are frequently administered. Moreover, to avoid polypharmacy, we propose to check the medication notebook. (Level: D, Grade: 2)

## Chapter 13. Dialysis initiation

CQ1: When should CKD patients be introduced to nephrologists to appropriately prepare for dialysis initiation?

Statement: It has been reported that the nephrologist’s care of CKD patients before their dialysis initiation affects their selection of kidney transplantation or dialysis, increases the probability of success of operation of vascular access, and decreases early mortality after their dialysis initiation. When patients are at least CKD Stage G4, their consultation with nephrologists is proposed. (Level: C, Grade: 2)

CQ2: To prevent the progression of kidney injury and late dialysis initiation, is patient education by various medical staff members recommended for CKD patients at CKD stage G3b or later?

Statement: Because interdisciplinary team management including doctors and nurses may prolong dialysis initiation, patient education by various medical staff members is recommended for CKD patients at CKD stage G3b or later. (Level: C, Grade: 2)

CQ3: Should we perform CVD screening at the initiation of dialysis?

Statement: There is no evidence to suggest that universal CVD screening is beneficial. However, as CKD is a well-known risk factor for CVD, we suggest treatable CVD should be identified at least before the initiation of dialysis. (Level: D, Grade: 2)

## Chapter 14. Kidney transplantation

CQ2: Is clinical follow-up recommended in post nephrectomy living kidney donors?

Statement: We suggest donors undergo appropriate follow-up similarly to CKD patients (Level: D, Grade: 2).

CQ2: Is preemptive kidney transplantation (PEKT) recommended?

Statement: We suggest that PEKT is the renal replacement modality of choice for all suitable patients whenever a donor is available. (Level: D, Grade: 2)

CQ3: Is PEKT recommended for diabetic patients?

Statement: We suggest that PEKT is the renal replacement modality of choice for diabetic patients. (Level: D, Grade: 2)

## Chapter 15. Management of drug treatment

CQ1: Is either an NSAID or acetaminophen recommended for patients with CKD suffering from pain?

Statement: Acetaminophen may be safer than NSAIDs for the short-term administration to patients with CKD. In particular, for elderly patients with decreased renal blood flow and GFR, the use of acetaminophen is proposed. However, even for acetaminophen, the safety of the long-term administration is not certain. (Level: D, Grade: 2)

CQ2: Is it recommended to treat patients with an oral spherical carbon adsorbent?

Statement: Although the therapeutic effect of an oral spherical carbon adsorbent on end-stage kidney disease (ESKD) or death in CKD patients is unclear, its treatment may be considered because there is a possibility of delaying CKD progression. (Level: C, Grade: 2)

CQ3: Is it recommended to reduce treatment with antiviral drugs based on renal function in CKD patients suffering from herpesvirus infection?

Statement: When administering antiviral drugs to CKD patients suffering from herpes simplex/herpes zoster virus infection, we propose designing drug administration based on evaluation of the renal function. In many cases, it may be expected that there will be a decrease in the adverse event occurrence by a drug administration design based on renal function, but even if only the dose is adjusted, it is necessary to pay close attention to the occurrence of adverse events (Level: D, Grade: 2).

CQ4: How does CKD affect anticoagulant treatment of patients with non-valvular atrial fibrillation?

Statement: Anticoagulant treatment for non-valvular atrial fibrillation associated with CKD patients may reduce the risk of total death and thromboembolism. Meanwhile, given the potential increased risk of developing severe bleeding complications, a decision for administration is made in considering the balance between benefits and disadvantages in each case. (Level: D, Grade: 2)

CQ5: Does iodinated radiocontrast media worsen kidney function in people with CKD?

Statement: All individuals with GFR < 60 mL/min/1.73 m^2^ (GFR categories G3a-5) are at risk for contrast media induced nephropathy (CIN) by the intravascular administration of iodinated radiocontrast media. This risk may increase especially in people with GFR < 45 mL/min/1.73 m^2^ (GFR categories G3b-5) or in cases in which large amount of iodinated radiocontrast media was used. (Level: B, Grade: 1)

If the radiography with intravascular administration of iodinated radiocontrast media is required for patients with GFR < 60 mL/min/1.73 m^2^ (GFR categories G3a-5), informed consent, sufficient hydration with saline before, during, and after the procedure, and use of lowest possible iodinated radiocontrast media dose are recommended. (Level: D, Grade: 1)

CQ6: Is it recommended prescribers should take the kidney function into account when renally excreted drugs in people with CKD?

Statement: We recommend that prescribers take kidney function into account and modify administration methods and doses of renally excreted drugs in patients with CKD to reduce the possibility of adverse events. (Level: C, Grade: 1)

## Chapter 16. Management of diabetic kidney disease

CQ1: Is the measurement of urine albumin recommended in a patient with diabetes mellitus?

Statement: Measurement of urine albumin is recommended in patients with diabetes mellitus since it is essential for the early diagnosis of diabetic nephropathy. (Level: B, Grade: 1)

CQ2: Is the administration of loop diuretics recommended for DKD patients with edema?

Statement: The administration of loop diuretics is recommended for DKD patients with excess fluids. However, concomitant use with RAS inhibitors or NSAIDs, or administration of excessive doses of loop diuretics may cause renal deterioration. Thus, careful follow-up observation is required throughout the administration period. (Level: D, Grade: 2)

CQ3: Is glycemic control with a target HbA1c level of less than 7.0% recommended in diabetic patients with chronic kidney disease?

Statement: Glycemic control aiming for an HbA1c level of less than 7.0% is recommended to prevent the progression of early stage diabetic nephropathy. There is not enough evidence supporting the benefits of intensive glycemic control on renal outcomes in patients with diabetes with macroalbuminuria or renal dysfunction. Caution is required for the risk of hypoglycemia when aiming for an HbA1c level of less than 7.0%. (Level: B, Grade: 1)

CQ4: Is a multifactorial intensive treatment recommended in patients with diabetes mellitus?

Statement: Multifactorial intensive treatment including a life-style modification and suitable management of risk factors such as blood glucose levels, BP and lipid profiles is recommended to inhibit the development and progression of diabetic vascular complications and to improve prognosis in patients with diabetes mellitus. (Level: B, Grade: 1)

## Chapter 17: Management of intractable kidney disease

17-1: Management of IgA Nephropathy

CQ1: Is family history associated with disease onset or progression in IgA nephropathy?

Statement: There is no evidence that family history is associated with disease onset or progression in IgA nephropathy at present. (Level: D, Grade: None)

CQ2: Does hematuria (macroscopic or microscopic) influence renal outcome in patients with IgA nephropathy?

Statement: Currently, there is no sufficient evidence indicating hematuria influences renal outcome in patients with IgA nephropathy. (Level: C, Grade: None)

CQ3: Are RAS inhibitors recommended for use in patients with IgA nephropathy?

Statement: Since it has been reported that ACEIs or ARBs decrease the incidence of ESKD, the progression of renal dysfunction and reduce the amount of proteinuria in patients with IgA nephropathy, use of RAS inhibitors is recommended. (Level: B, Grade: 1)

17-2: Management of Nephrotic Syndrome

CQ1: Are RAS inhibitors recommended for treatment of steroid-resistant or immunosuppressant-resistant primary nephrotic syndrome without hypertension in adults?

Statement: In adults with steroid-resistant or immunosuppressant-resistant primary nephrotic syndrome but without hypertension, we suggest using ACEIs or ARBs (not covered by medical insurance). (Level: C, Grade: 2)

CQ2: Is diagnosis by renal biopsy recommended for elderly patients with nephrotic syndrome?

Statement: We found no evidence to suggest/recommend renal biopsy in elderly patients with nephrotic syndrome and we propose consultation with kidney specialists and special medical institutions for specific indications. (Level: D, Grade: None)

CQ3: Is restriction of daily activity or exercise recommended for the patients with nephrotic syndrome?

Statement: We found no evidence of benefit or harm from restricting daily activity or exercise for long-term outcomes in patients with nephrotic syndrome. (Level: D, Grade: None)

17-3: Management of Autosomal Dominant Polycystic Kidney Disease (ADPKD)

CQ1: Should patients with ADPKD be screened for intracranial aneurysms?

Statement: There is no direct evidence to demonstrate that screening for intracranial aneurysms is beneficial for lowering overall mortality or preventing rupture of aneurysms. Considering that prevalence of intracranial aneurysms in ADPKD patients is significantly higher than that in general population, however, their screening using MRI is suggested for ADPKD patients with a family history of intracranial aneurysms or subarachnoid hemorrhage. (Level: D, Grade: 2)

CQ 2: Is tolvaptan recommended for ADPKD patients?

Statement: We recommend using tolvaptan for autosomal dominant ADPKD patients with a total kidney volume of ≥ 750 mL and with an estimated creatinine clearance of ≥ 60 mL/min. We also recommend using tolvaptan for later-stage ADPKD patients with an eGFR of 25–60 mL/min/1.73 m^2^ to slow the increase in total kidney volume and the decline in kidney function. Safety and efficacy of tolvaptan in children have not been established yet. (Level: B, Grade: 1)

CQ3: Can RAS inhibitors be administered for hypertensive ADPKD patients?

Statement: We suggest that ACEIs or ARBs should be administered for hypertensive ADPKD patients from the viewpoint of suppressing the development of ESKD. (Level: C, Grade: 2)

17-4: Management of Rapidly Progressive Glomerulonephritis (RPGN)

CQ1: Is the use of “The guide for early diagnosis of rapidly progressive nephritic syndrome” recommended for detecting RPGN in patients with urinary abnormalities and renal dysfunction?

Statement: We recommend the use of “The guide for early diagnosis of rapidly progressive nephritic syndrome” for detecting RPGN in patients with urinary abnormalities and renal dysfunction. (Level: D, Grade: 1)

CQ2: Is testing serum MPO-ANCA, PR3-ANCA and anti-GBM antibody levels recommended for a differential diagnosis of RPGN in patients showing urinary abnormalities and worsening renal function within a few weeks or a few months?

Statement: Immediate testing of serum MPO-ANCA, PR3-ANCA and anti-GBM antibody levels is recommended for a differential diagnosis of RPGN in patients showing urinary abnormalities and worsening renal function. (Level: D, Grade: 1)

CQ3. Is starting glucocorticoids alone useful for the initial treatment of patients with ANCA-associated RPGN?

Statement: For the initial treatment of ANCA-associated RPGN, starting glucocorticoids alone is useful and is suggested for ameliorating patient prognosis. (Level: D, Grade: 2)

However, we recommend that patients refractory to initial treatment or having organ-threatening or life-threatening involvement be referred to, or managed in close collaboration with, an expert center and combined use of immunosuppressive therapy should be considered. (Level: D, Grade: 1)

## List of Contributors

**Committee of Evidence-based Practice Guideline for the Treatment of CKD 2018**

**Chair**

Hirokazu Okada: Department of Nephrology, Saitama Medical University

**Co-Chair**

Yoshinari Yasuda: Department of CKD Initiatives, Nagoya University Graduate School of Medicine

**President, Japanese Society of Nephrology**

Naoki Kashihara: Department of Nephrology and Hypertension, Kawasaki Medical School

**Board Members**

Koichi Asahi: Division of Nephrology and Hypertension, Department of Internal Medicine, Iwate Medical University School of Medicine

Takafumi Ito: Division of Nephrology, Shimane University Hospital
Shinya Kaname: Division of Nephrology and Rheumatology, The First Department of Internal Medicine, Kyorin University School of Medicine
Eiichiro Kanda: Medical Science, Kawasaki Medical School
Yoshihiko Kanno: Department of Nephrology, Tokyo Medical University
Kenichi Shikata: Center for Innovative Clinical Medicine, Okayama University Hospital
Yugo Shibagaki: Division of Nephrology and Hypertension, St Marianna University School of Medicine
Ken Tsuchiya: Department of Blood Purification Tokyo Women's Medicl University
Kazuhiko Tsuruya: Department of Nephrology, Nara Medical University

Daisuke Nagata: Division of Nephrology, Department of Internal Medicine, Jichi Medical University
Ichiei Narita: Division of Clinical Nephrology and Rheumatology, Niigata University Graduate School of Medical and Dental Sciences
Masaomi Nangaku: Division of Nephrology and Endocrinology, the University of Tokyo Graduate School of Medicine
Motoshi Hattori: Department of Pediatric Nephrology, Tokyo Women's Medical University
Takayuki Hamano: Department of Inter-Organ Communication Research in Kidney Disease, Osaka University Graduate School of Medicine
Shouichi Fujimoto: Department of Hemovascular Medicine and Artificial Organs, Faculty of Medicine, University of Miyazaki
Toshiki Moriyama: Health and Counseling Center, Osaka University
Kunihiro Yamagata: Department of Nephrology, Faculty of Medicine, University of Tsukuba
Ryohei Yamamoto: Health and Counseling Center, Osaka University
Minako Wakasugi: Division of Comprehensive Geriatrics in Community, Niigata University Graduate School of Medical and Dental Sciences
Akira Ashida: Department of Pediatrics, Osaka Medical College
Joichi Usui: Department of Nephrology, Faculty of Medicine, University of Tsukuba
Kazuko Kawamura: Division of Clinical Nephrology and Rheumatology, Kidney Research Center, Niigata University Graduate School of Medical and Dental sciences
Kenichiro Kitamura: Third Department of Internal Medicine, University of Yamanashi School of Medicine
Tsuneo Konta: Department of Public Health and Hygiene, Yamagata University Graduate School of Medical Science
Yusuke Suzuki: Department of Nephrology, Juntendo University Graduate School of Medicine
Shuichi Tsuruoka: Department of Nephrology, Nippon Medical School
Saori Nishio: Division of Rheumatology, Endocrinology and Nephrology, Hokkaido University Graduate School of Medicine
Takayuki Hamano: Department of Inter-Organ Communication Research in Kidney Disease, Osaka University Graduate School of Medicine
Naohiko Fujii: Department of Nephrology, Hyogo Prefectural Nishinomiya Hospital
Hideki Fujii: Division of Nephrology and Kidney Center, Kobe University Graduate School of Medicine
Takehiko Wada: Division of Nephrology, Endocrinology and Metabolism, Tokai University School of Medicine

**Adviser**

Hitoshi Yokoyama: Kanazawa Medical University School of Medicine, Department of Nephrology

**Evidence-based Practice Guideline for the Treatment of CKD Production Group (Work group)**

Katsunori Aoki: Department of Nephrology Osaka University Graduate School of Medicine
Daiichiro Akiyama: Third Department of Internal Medicine, University of Yamanashi School of Medicine
Shin-ichi Araki: Division of Diabetology, Endocrinology and Nephrology, Department of Medicine, Shiga University of Medical Science
Hisatomi Arima: Department of Preventive Medicine and Public Health, Fukuoka University
Eiji Ishikawa: Department of Cardiology and Nephrology, Mie University Graduate School of Medicine
Kenji Ishikura: Division of Nephrology and Rheumatology, National Center for Child Health and Development
Kiyonobu Ishizuka: Department of Pediatric Nephrology, Tokyo Women's Medical University, School of Medicine
Takuji Ishimoto: Nagoya University Hospital Department of Nephrology
Yu Ishimoto: (1) Division of Nephrology and Endocrinology, The University of Tokyo, Graduate School of Medicine (2) Division Chronic Kidney Disease Pathophysiology, The University of Tokyo, Graduate School of Medicine
Kunitoshi Iseki: Nakamura Clinic, Clinical Research Support Center
Mitsuyo Itabashi: Department of Nephrology, Tokyo Metropolitan Geriatric Hospital
Satoko Ichioka: Department of Pediatrics, Shiga University of Medical Science
Kazunobu Ichikawa: Department of Cardiology, Pulmonology, and Nephrology, Yamagata University School of Medicine
Daisuke Ichikawa: Division of Nephrology and Hypertension, Department of Internal Medicine, St Marianna University School of Medicine
Shuji Inoue: Department of Nephrology, Saitama Medical University
Toshimi Imai: Division of Nephrology, Department of Internal Medicine, Jichi Medical University
Hideaki Imamura: Division of Pediatrics, Department of Developmental and Urological-Reproductive Medicine, Faculty of Medicine, University of Miyazaki
Yasunori Iwata: Kanazawa University Hospital, Division of Nephrology
Yoshitaka Iwazu: Clinical Laboratory Medicine & Department of Nephrology, Jichi Medical University
Toshiaki Usui: Department of Nephrology, Faculty of Medicine, University of Tsukuba
Keiko Uchida: Hearth Administration Center, Tokyo Women's Medical University
Masahiro Egawa: Department of Nephrology, Shimane University Hospital
Shinichiro Ohara: Ohara Children Clinic
Norio Omori: Tokyo Metropolitan Children's Medical Center Department of Nephrology
Rieko Okada: Department of Preventive Medicine, Nagoya University Graduate School of Medicine
Yusuke Okuda: Shiga University of Medical Science
Takaya Ozeki: Department of Nephrology, Nagoya University Graduate School of Medicine
Yoko Obata: Department of Nephrology, Nagasaki University Hospital
Hirayasu Kai: Department of Nephrology, Faculty of Medicine, University of Tsukuba
Noritoshi Kato: Nagoya University Hospital Department of Nephrology
Keizo Kanasaki: Department of Diabetology & Endocrinology, Kanazawa Medical University
Yoshikatsu Kaneko: Division of Clinical Nephrology and Rheumatology, Niigata University Graduate School of Medical and Dental Sciences
Hideyuki Kabasawa: Department of Clinical Nutrition Science, Kidney Research Center, Niigata University Graduate School of Medical and Dental Sciences
Takehiko Kawaguchi: Department of Nephrology, Chiba East Hospital
Yukihiko Kawasaki: Department of Pediatrics, Fukushima Medical University School of Medicine
Keisuke Kawashima: Division of Rheumatology, Endocrinology and Nephrology, Hokkaido University Graduate School of Medicine
Haruna Kawano: Department of Urology, Juntendo Graduate School of medicine
Kan Kikuchi: Shimoochiai Clinic
Masao Kihara: Department of Nephrology, Juntendo University Faculty of Medicine
Yoshiki Kimura: Department of Nephrology, Osaka University Graduate School of Medicine
Noriaki Kurita: Department of Innovative Research and Education for Clinicians and Trainees, Fukushima Medical University Hospital
Kentaro Koike: Division of Nephrology and Hypertension, Department of Internal Medicine, The Jikei University School of Medicine
Masahiro Koizumi: Division of Nephrology, Endocrinology, and Metabolism, Tokai University School of Medicine
Chiari Kojima: Department of Nephrology, Saitama Medical University
Shunsuke Goto: Division of Nephrology and Kidney Center, Kobe University Graduate School of Medicine
Takao Konomoto: Division of Pediatrics, Department of Developmental and Urological-Reproductive Medicine, Faculty of Medicine, University of Miyazaki
Kentaro Kohagura: Dialysis Unit, University of the Ryukyus Hospital
Hiroyuki Komatsu: Center for Medical Education and Career Development Faculty of Medicine, University of Miyazaki
Hirotaka Komaba: Division of Nephrology, Endocrinology and Metabolism Tokai University School of Medicine
Chie Saito: Department of Nephrology, Division of Clinical Medicine, Faculty of Medicine, University of Tsukuba
Yukinao Sakai: Department of Nephrology, Graduate School of Medicine, Nippon Medical School
Yusuke Sakaguchi: Department of Inter-Organ Communication Research in Kidney Disease, Osaka University Graduate School of Medicine
Hiroshi Satonaka: Department of Cardiology and Nephrology Dokkyo Medical University
Kanako Jimi: Department of Nephrology, Tokyo Medical University
Akihiro Shimizu: Division of Nephrology and Hypertension, Department of Internal Medicine, The Jikei University School of Medicine
Sayaka Shimizu: Department of Healthcare Epidemiology, School of Public Health in the Graduate School of Medicine, Kyoto University
Sayuri Shirai: Department of Nephrology and Hypertension St. Marianna University School of Medicine Yokohama City Seibu Hospital
Maki Shinzawa: Department of Nephrology　Graduate School of Medicine Osaka University
Kazuhiro Sugiyama: Internal medicine department of nephrology Tokoname Tokoname City Hospital
Tomo Suzuki: Division of Nephrology and Hypertension, Department of Internal Medicine, St. Marianna University School of Medicine
Hitoshi Suzuki: Department of Nephrology, Juntendo University Faculty of Medicine
Kazuhide Suyama: Department of Pediatrics, Fukushima Medical University School of Medicine
Hiroyoshi Segawa: Center for Epidemiologic Research in Asia Leading Graduate Program, Shiga University of Medical Science
Kazuya Takahashi: Third Department of Internal Medicine, University of Yamanashi School of Medicine
Kenichi Tanaka: Department of Nephrology and Hypertension, Fukushima Medical University
Tetsuhiro Tanaka: Division of Nephrology and Endocrinology, the University of Tokyo School of Medicine
Ryoya Tsunoda: Department of Nephrology, Unversity of Tsukuba Hospital
Yuki Tsuruta: Tsuruta Itabashi Clinic
Hyogo Nakakura: Department of Hemodialysis and Aphresis in Arisawa General Hospital
Yasuyuki Nagasawa: Hyogo College of Medicine, Department of Internal Medicine, division of Kidney and Dialysis
Koichi Nakanishi: Department of Child Health and Welfare (Pediatrics), Graduate School of Medicine, University of the Ryukyus
Masahiko Nagahama: Division of Nephrology St. Luke’s International Hospital
Izaya Nakaya: Department of Nephrology and Rheumatology, Iwate Prefectural Central Hospital
Masayoshi Nanami: Department of Internal Medicine, Division of Kidney and Dialysis, Hyogo College of Medicine
Kakuya Niihata: Department of Hygiene and Preventive Medicine, Fukushima Medical University School of Medicine
Shinichi Nishi: Division of Nephrology and Kidney Center, Kobe University Graduate School of Medicine
Hiroki Nishiwaki: Division of Nephrology, Department of Medicine, Showa University Fujigaoka Hospital
Shoko Hasegawa: Department of Medicine and Clinical Science, Graduate School of Medical Sciences Kyushu University
Midori Hasegawa: Fujita Health University School of Medicine, Department of Nephroloty
Ken Hanada: Division of Clinical Rheumatology and Nephrology, Matsue Red Cross Hospital
Hiroki Hayashi: Department of Nephrology, Fujita Health University School of Medicine
Ryoko Harada: Tokyo Metropolitan Children's Medical Center Department of Nephrology
Manabu Hishida: Department of Nephrology, Graduate School of Medicine, Nagoya University
Daishi Hirano: The Jikei University School of Medicine
Junichi Hirahashi: Center for General Medicine Education, Keio University School of Medicine
Akio Hirama: Nippon Medical School Hospital
Kouichi Hirayama: Department of Nephrology, Tokyo Medical Univsersity Ibaraki Medical Center
Masafumi Fukagawa: Division of Nephrology, Endocrinology, and Metabolism Tokai University School of Medicine
Akihiro Fukuda: Department of Endocrinology, Metabolism, Rheumatology and Nephrology, Faculty of Medicine, Oita University
Yoshiyuki Fujii: Department of Nephrology, Osaka University Graduate School of Medicine
Kiichiro Fujisaki: Department of Nephrology, Hypertension and Storokology, Kyushu University Hospital
Fumihiko Furuya: Third Department of Internal Medicine, University of Yamanashi School of Medicine
Junichi Hoshino: Nephrology Center, Toranomon Hospital, Tokyo
Michihiro Hosojima: Department of Clinical Nutrition Science, Kidney Research Center, Niigata University Graduate School of Medical and Dental Sciences
Kenjiro Honda: Department of nephrology and endocrinology, The University of Tokyo
Takahiro Masuda: Division of Nephrology, Jichi Medical University
Kosuke Matsui: Department of Nephrology, Heisei Memorial Hospital
Yuta Matsukuma: Department of Medicine and Clinical Science, Kyushu University
Hideki Matsumura: Department of Pediatrics, Osaka Medical College
Akiko Mii: Department of Nephrology, Graduate School of Medicine, Nippon Medical School
Kenichiro Miura: Department of Pediatric Nephrology, Tokyo Women's Medical University
Michihiro Mitobe: The Takeda Healthcare Foundation Takeda General Hospital Nephrology
Yoshikazu Miyasato: Department of Nephrology, Graduate School of Medical Sciences, Kumamoto University
Satoshi Miyamoto: Center for Innovative Clinical Medicine, Okayama University Hospital
Saori Miwa: The Jikei University School of Medicine
Masahiko Yazawa: Department of Nephrology and Hypertension, St. Marianna University School of Medicine
Yusuke Yata: Saiseikai Niigata Daini Hospital
Yoshihiro Yamamoto: Toyota Memorial Hospital
Kimio Watanabe: Tohoku University Hospital, Division of Blood Purification
Michihiro Hosojima: Department of Clinical Nutrition Science, Kidney Research Center, Niigata University Graduate School of Medical and Dental Sciences

## Abbreviations

ACEI: Angiotensin converting enzyme inhibitor
AKI: Acute kidney injury
ARB: Angiotensin receptor blocker
BMI: Body mass index
BP: Blood pressure
CCB: Calcium channel blocker
CKD: Chronic kidney disease
CVD: Cardiovascular disease
eGFR: Estimated glomerular filtration rate
ESKD: End-stage kidney disease
MRB: Mineralocorticoid receptor blocker
QOL: Quality of life
RAS: Renin angiotensin system

